# Green Infrastructure and Urban-Renewal Simulation for Street Tree Design Decision-Making: Moderating Demands of Stormwater Management, Sunlight and Visual Aesthetics

**DOI:** 10.3390/ijerph19138220

**Published:** 2022-07-05

**Authors:** Nano Langenheim, Marcus White

**Affiliations:** 1 Melbourne School of Design, University of Melbourne, Masson Rd, Parkville, VIC 3010, Australia; nano.langenheim@unimelb.edu.au; 2 School of Design and Architecture, Swinburne University of Technology, John Street, Hawthorn, VIC 3122, Australia

**Keywords:** green infrastructure, urban design, street trees, stormwater management, nature-based solutions, simulation, shade modelling, design decision-support, procedural modelling, visualization

## Abstract

The design of green infrastructure in urban renewal sites is complex, requiring engagement with existing communities and future sustainable development goals, consideration of existing and future urban forms, changing climatic conditions, and the sites often being in low-lying and flood-prone areas. Traditional street tree decision-making approaches are inadequate for addressing the scale, environmental complexity, and mutability of decisions involved in urban renewal projects—new tree selection approaches that consider complex competing criteria for tree selections addressing stormwater management systems, visual assessment and solar amenity are needed. This paper describes a new method of multi-criteria street design decision modelling that combines outputs from hydrology modelling, digital procedural tree modelling and urban form analysis, with animation and gaming technologies. We evaluate our approach through application to the design of a large-scale, urban renewal project underway in Melbourne, Australia. The results of the study demonstrate the functionality of our model, which allowed the simultaneous output of streetscape visualisation, with tree selection responding to integrated stormwater management infrastructure and flooding, along with the likely overshadowing conditions of urban renewal built-form. Our multi-criteria approach makes a significant contribution to the tools available to urban designers, planners and landscape architects in their pursuit of smarter streetscape design decisions that respond to complex spatial, cultural and climatic urban challenges.

## 1. Introduction

Urban form, commonly referred to as urban morphology, or the urban ensemble, is a result of interactions between past planning decisions, building, infrastructure, road networks, and existing conditions [1,2,3,4], reflecting changes in environmental, cultural, social and economic circumstances over time [5,6]. For instance, Colonial urban developments, driven by popular planning paradigms of the 18th century, such as French formalism, exemplified in Barron von Haussmann’s renovation of the city of Paris [7], often have wide straight gridded streets with symmetrical tree plantings [8]. Subsequent evolution of these developments during the early 20th century saw these same streets inundated with roadside and median carparking, driven by a need to accommodate the rise of private motorised transport [9]. Today these urban developments are again evolving, driven by contemporary concerns and goals for sustainability and moderation of the impacts of climate change [10,11].

These contemporary concerns and goals are diverse, and complex, ranging from reduction of urban heat, reduction of stormwater flooding, reduction of arable land development and reduction of car dependence. Often, to meet these goals, urban renewal projects, which repurpose or ‘renew’ ex-industrial greyfield sites close to central business districts for high-density housing, are proposed. To successfully meet these goals, urban renewal projects require substantial departures from traditional, past urban norms [2,12,13,14,15,16].

The design and planning of sustainable urban renewal is challenging [17]. Interactions between previous land uses, existing unfavourable environmental conditions such as flooding, proposals for multistorey higher density-built form, and often limited quantity of publicly owned land, put pressure on the street network to accommodate not only transport networks, but also climate-sensitive green infrastructure [18,19]. In addition, traditional aesthetic design, or visual ‘attractiveness’, a key ingredient of walkable cities, must still be considered [20,21,22,23,24,25,26]. Often, these competing performance criteria converge in decisions about street trees.

Strategic selection, placement and spacing of street trees can support urban climatic performance in aspects such as heat moderation and storm water control, and support sustainable development goals by increasing the thermal comfort and visual attractiveness of streets for active transport [27,28,29]. Urban renewal projects often require contract growing of several thousand new street trees, and thus present a unique opportunity to strategically select and place trees in locations that meet both visual and environmental performance criteria. However, integrated tools and modelling methods that support tree selection and placement, which respond to built form variables, climate conditions, and traditional visual, aesthetic performance criteria are lacking [30,31]. Currently, the environmental and visual performance of tree planting proposals are modelled in ‘decoupled’, disciplinary silos. Environmental performance is modelled by environmental engineers while visual performance is modelled by landscape and urban designers. Integration of their outputs is limited and communication of environmental performance outcomes in formats suitable for community consultation forums is scant [32,33,34,35,36].

To address this challenge, we developed a visual-functional street tree-decision support approach and evaluated it through application to an urban renewal proposal test case in Melbourne, Australia. The approach builds on a previous tree decision-support model by the authors that integrates tree visual and shade performance. This enhanced model adds two new environmental performance criteria, a protection from winter over-shadowing component and a stormwater control measures component.

In this paper, we provide a brief background and limitations of current tree decisions and modelling methods for Storm Water Control Measures (SCM) (Section 2), seasonal variation of sunlight (Section 3) and visual aesthetic considerations (Section 4). We then put forward our aim and method for a new approach for green infrastructure and urban-renewal simulation for street tree decision-making that can be used to moderate between stormwater management, sunlight and visual aesthetic demands (Section 5). Finally, we describe the results of the application of our method on a test case, applying the approach to a complex urban renewal study site in Melbourne, Australia, (Section 6), and discuss the outcomes (Section 7 and Section 8).

## 2. Background to Street Trees in Storm Water Control Measures (SCM)

### 2.1. Choosing Species That Contribute to SCM Performance

Urban renewal projects are often located on flood-prone land close to waterways at the base of hydrological catchments, as proximity to water was a critical consideration for their past, often industrial, land use. The existing drainage infrastructure is often inadequate for servicing higher residential densities and the greater intensity rainfall events associated with climate change [37,38,39]. Green infrastructure, particularly storm water control measures (SCM), such as biofiltration systems, water-sensitive tree pits, detention ponds and rain gardens, can be used to support this drainage network [38,39,40,41,42]. Many of these SCM have considerable spatial requirements. On project sites with little public open space, SCM must be realised in streetscapes, substantially impacting street design. Two primary forms of streetscape SCM are roadside detention ponds (road-based integrated water management), and ‘Land Subject to Inundation’ (LSIO) overlays, that dictate special adjustments to the ground-level design of buildings [13,43]. Critical to the success of both of these measures is the selection of planting within them, particularly the choice of tree species [41].

### 2.2. SCM Modelling Methods

Modelling and mapping can be used to visualise the likely extent of flood events in existing urban form for LSIO overlays. While modelling of the extent of stormwater flooding for existing conditions is complex as both the overland flow and the underground pit and pipe system must be considered, modelling the impact of SCM design options considering future urban form proposals is exponentially more so [44,45,46,47]. New methods are evolving that allow flood modelling professionals to test the impact of major reconfigurations of street casements to include storm water detention using a modified street section approach and discussed later in this paper.

Recently, the outputs of SCM design models have been coupled with models for quantifying the impact of different tree type seasonal variation (evergreen or deciduous) and percentages of canopy cover, the most notable of these being the Urban Multiscale Environment Predictor (UMEP) [48] and the USDA’s iTrees Hydro model [45,49]. These models generally reveal that from a purely SCM perspective, it is best to maximise plantings of large dense-foliaged evergreen species (such as conifers) and those with constant rates of transpiration, particularly in locations where winter rainfall rates are high. These species can improve the performance of SCM by either slowing rates of overland flow through canopy rainfall interception, or absorbing stormwater held in detention in biofiltration systems, through evapotranspiration [45,50,51].

### 2.3. Street Tree SCM Considerations

Though SCM-focused street tree-decision modelling may strongly suggest elimination of deciduous trees in favour of evergreen trees, this choice would exacerbate temperate climate, higher density urban development problems of deep winter overshadowing [52] and would conflict with known community visual aesthetic preferences [53]. It is therefore important to balance streetscape tree selection criteria for SCM with other environmental and visual criteria.

## 3. Background to Seasonal Sunlight Variation

### 3.1. Avoiding Oppressive Solar Exposure in Summer and Gloomy Overshadowing in Winter

**Figure 1:**
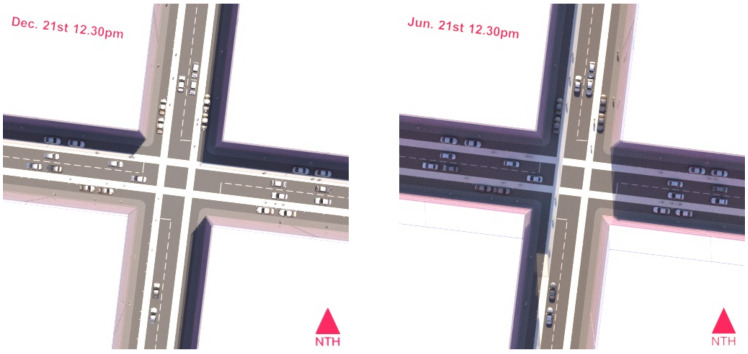
An intersection of a street with equal building height to street width ratio. LEFT 12:30 in summer; RIGHT 12:30 in winter. Higher density can still leave pedestrians exposed in summer; on this orientation, winter solar access could be preserved on the western side of north–south oriented streets. Image by authors using Autodesk™ 3ds Max™.

Depending on urban canyon geometry (street width to building height ratio) and street orientation, higher density developments in temperate climates can have hot, solar-exposed streets in summer when sun angles are high, and gloomy overshadowed solar-deprived streets in winter when sun angles are low (see Figure 1), making them uncomfortable pedestrian environments most of the year [30,52,54,55,56]. Summer pedestrian and cyclist comfort can be improved by maximising tree shade through selection of species with spreading canopies, planted at spacings that allow the canopy cover to be continuous. Winter comfort can be improved by maximising the use of deciduous trees that lose their leaves in winter, in locations that fall beyond the winter building shadow extents [57,58,59].

### 3.2. Modelling Seasonal Sunlight Variation Interaction with Proposed Built Form

One of the most substantial barriers to understanding the future light conditions of urban renewal projects is that size and height regulations for buildings are often specified in a written (non-spatial) planning guideline format. These regulations are also often driven by considerations such as historic norms, market forces, street activation requirements and density targets rather than environmental performance [15].

These written regulations are difficult to include in models run for other purposes such as ecosystem service calculation, and thus most environmental modelling methods, developed to quantify the interactions between sunlight, urban tree canopies and built form [3], focus on the analysis of existing urban form rather than proposed form. In addition, these models tend to focus on quantification of tree benefits in summer heat stress reduction, rather than their disbenefits such as contributions to winter overshadowing, exacerbation of ‘right to light’ issues and human health impacts caused by lack of access to sunlight [60,61,62].

Recent attempts to couple tree impact calculation with proposed urban form scenarios, including the thermal comfort mapping approach by Coccolo et al. [63], the iTree ecosystem service calculation integration with scenario planning by Hilde and Paterson [64], the aspect ratio and street orientation approach to calculating thermal comfort by Ali-Tourdet and Mayer [65] and the urban assembly method developed by Lee and Mayer, have shown great promise. However, these models are still focused primarily on summer tree benefit calculation rather than cooler season overshadowing problems. In addition, the outputs of these models represent trees as abstract forms such as voxels (Envi_MET) or lollipops (ball on stick) rather than a visually realistic geometry, thus limiting their suitability for use by planning and design professions, particularly when making tree decisions in conjunction with the community [57,66].

### 3.3. Modelling for Different Criteria in Disciplinary Silos

While there are emerging modelling approaches capable of improving environmentally responsive tree decision-making, there are still problems with their adoption and implementation [67]. Each model is complex, each is predominantly single criteria, and all are undertaken using vastly different software platforms. Systems in urban environments are interactive and multicriteria; building form and street orientation must inform tree placement and species selection for winter and summer active transport comfort, while tree selections for flood moderation must inform building setbacks to allow space for SCM. In addition, both building planning regulations and environmental modelling outputs are decoupled from traditional street design visualisation techniques used by designers negotiating tree decisions with local resident groups and communities [33,35]. This is important to recognise, as while trees can be used to improve the environmental performance of development, this has not been their traditional basis for inclusion on urban streets [27,68,69].

## 4. Background to Visual Consideration of Trees in Streets

### 4.1. Street Tree Visual Aesthetic Considerations

Proportion, arrangement of forms, and colours, as first mentioned in Roman architect Marcus Vitruvius Pollio’s ‘Vitruvian trilogy’ of ‘*firmitas, utilitas, et venustas*’ or ‘firmness, commodity and delight’ in *De architectura*, referred to as *The Ten Books on Architecture*, have long been core measures of successful design and placemaking [70,71]. As such, architecture and urban design visualisations have been an integral component of the design process, used by designers to test design concepts [72] and to communicate these with clients or stakeholders [73,74,75,76].

Street trees have played an important visual role in the urban design of new or renewed cities since the 1800s, profoundly influencing the quality and attractiveness of streetscapes. Street trees can provide a ‘sense of safety and place’, ‘create a sense of streetscape enclosure’, ‘define the street as an outdoor room’, ‘distinguish a boundary between different street uses’ and support a ‘sense of civic identity’ [68,77,78]. Street tree planting has traditionally been driven by visual and symbolic principles, arranged in evenly spaced symmetrical rows of homogenous species, reflecting the influence of the *Beaux*-*Arts* French Formal compositional ideas on urban design during the expansion of the British Colonial Empire [57,79,80,81]. This compositional importance of street trees for landscape architects, urban designers and the community has persisted to this day with ‘street attractiveness’ proven to be a significant factor in choosing active travel (walking and cycling) [20,22,23,25,26].

While the importance of streetscape ‘attractiveness’ has not diminished in recent decades, the shifting focus of tree selection principles to prioritise environmental performance requires a reassessment of symmetrical mono-species urban tree-scapes. In higher-density urban development, asymmetric light conditions occur due to interactions between buildings, street orientation and sun angles, which might require smaller trees on the ‘shady side’, to minimise any additional overshadowing in winter and larger trees on the ‘sunny side’ to maximise summer shade [57,82]. Without careful communication and visualisations, tree decisions such as this might attract community resistance and low levels of support [52,83,84,85].

### 4.2. Modelling Methods for Street Tree Visual Aesthetic Considerations

In current practice, methods for making tree selections in urban renewal are predominantly undertaken in 2D section, plan and collage or photomontage formats. These images are efficient to produce, highly effective for assessing visual outcomes and useful in community consultation environments, where concerns are likely to centre on visual and cultural preferences and historic or traditional preferences [57,86,87].

**Figure 2:**
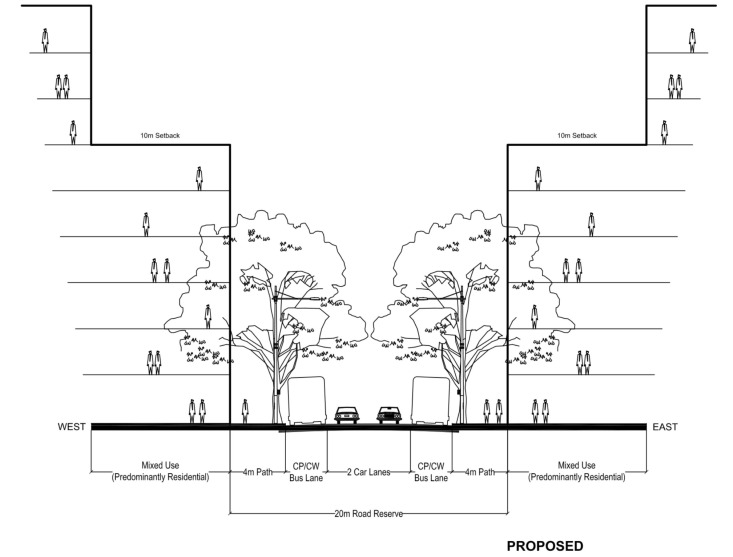
Proposed design for the connector road (Boundary Road). Source: Arden Macaulay Structure plan 2012. This drawing shows the proposed street wall (zero lot building façade) and the conflict it has with the canopies of proposed trees.

These 2D methods of streetscape design representation are, however, not suitable for testing decisions, against either environmental performance or the written guidelines of built form regulations. In more complex urban morphologies, its use has led to conflicts of canopies with overhead powerlines and building facades during implementation (see Figure 2) [88].

3D tree simulation is an area of research for many disciplines from the gaming industry, defence, hydrology catchment management to agroforestry [89,90]. While the uses of digital tree models differ between disciplines, many are constructed from the same recursive branching methods developed by Honda [91] to provide visually and structurally realistic three-dimensional, polygon-dense tree models [90]. While it was not initially feasible to use these 3D high polygon trees in urban scaled precinct and streetscape design decision-making models due to the required computing power, recent advances in software and hardware have improved this situation [90].

### 4.3. How Visually Accurate Tree Models Assist Environmental Performance Modelling

**Figure 3:**
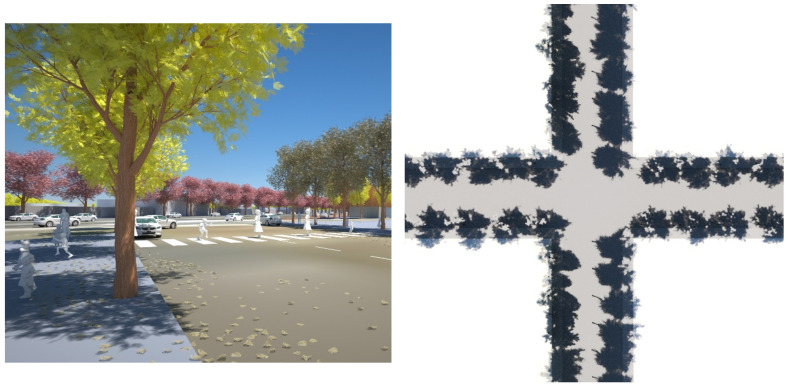
Image showing visual aesthetic assessment with high-definition streetscape rendered visualisation LEFT and texture baked shade assessment output RIGHT. Image by authors using multi-criteria street design decision support modelling approach in Autodesk™ 3ds Max™.

In recent work, we combined these 3D high polygon tree models within a precinct model, to develop a performance-based visual-functional street design approach [57,90]. That model outputs both quantitative shade calculations and traditional qualitative visual impact imagery simultaneously (Figure 3). The model used gaming and animation industry software, an accurate solar metric sun-system, advanced GPU-enabled processing and a texture baking technique that captures shadows of objects in isolation (tree shadows), on a surface [92]. While the model showed promising possibilities for the use of 3D high-polygon tree models for simultaneously visual and environmentally responsive design, it did not engage with flooding or the complexities of large-scale urban renewal proposals and the winter streetscape overshadowing this can generate.

Selecting trees to address the criteria of SCM, summer thermal comfort and winter light access, all while offering minimal disruption to aesthetic principles in higher density urban renewal projects, is a challenging problem that requires balance and compromise. Development of performance-based design approaches that intersect traditional visualisation with ecosystem service calculation and spatially explicit decision analysis is critical [57,93]. To our knowledge, there are currently no tools that integrate simple climatic performance with production of high-quality visual assessment materials. For urban renewal tree decisions that must moderate between these competing criteria, there is a clear gap in currently available approaches. To address this gap, a street tree design decision-making approach needs to include the following:

1. Integrate and assess existing and proposed urban form (building massing) for shadow and visualisation;
2. Integrate different proposed approaches to storm water control measure (SCM) modelling and subsequent flood inundation implications;
3. Link flood inundation area maps with appropriate tree species choices for projected flood conditions (flood tolerant or non-flood tolerant species for in-flood and not-in-flood conditions);
4. Calculate shadow projections for different times of day and different days of the year;
5. Link projected shadow maps of built urban form with appropriate tree species choices for projected shadow conditions to maximise tree-shade in summer and minimise tree-shade in winter (evergreen or deciduous for in-shade and not-in-shade conditions);
6. Provide rapid visualisation of light levels of streetscape designs;
7. Provide rapid visualisation of streetscape condition-responsive street tree designs with 3D trees for visual aesthetic consideration.

## 5. Method: A Multi-Criteria Street Design Decision Support Modelling Approach

Our aim was to develop and test a green infrastructure and urban-renewal simulation for street tree decision-making that moderates between the demands for stormwater management, sunlight and aesthetics.

The method for this study builds on the Spatio-temporal Design Decision Support System approach by White and Langenheim, expanding their previous model for assessing solar exposure in existing built form conditions for both visual analysis and tree shade quantification [57,90].

The model employs the functionality of gaming and animation industry software (Autodesk 3dsMax™) with a solar metric sun-system and advanced processing of three-dimensional polygon-dense ‘proxy object’ three-dimensional trees (a ‘proxy-object’ is a highly efficient modelling method used in the animation industry for scattering hundreds or even thousands of the same complex object (with procedural translations such as scale and rotation to reduce the look of repetition) to simulate large scenes such as forests).

This model comprises five components:

(1) Spatializing built form planning controls as an accurate 3D (precinct) model.
(2) Utilizing the solar metric sun system within the precinct model to assess both summer solar exposure and winter overshadowing of streets (proposed and retained built form only), output as conditions or ‘presence’ maps.
(3) Integration of flood modelling output as ‘presence of’/‘absence of’ conditions maps.
(4) Selection of indicative tree species suitable for intersections of conditions or co-conditions (i.e., species tolerant of flooding and overshadowing and species tolerant of flood and that minimise additional streetscape overshadowing in winter).
(5) Use of 3D high polygon visually accurate tree models to represent the above species selection in the precinct model, with their occurrence in the model controlled by the ‘presence of’/‘absence of’ conditions or co-conditions maps.

This same model can be used to generate shade maps that are ‘rendered out’, using a texture baking technique that captures shadows on a surface [92]. These shadow maps are then combined with flood inundation maps to generate ‘presence–absence’ flooding and sunlight conditions maps. We then use these ‘presence–absence’ maps to procedurally drive the selection of tree species based on the specific flooding and light condition.

To test the effectiveness, we have applied our approach on a test case in inner Melbourne, Australia. We chose a complex major urban renewal project with distinct flooding as well as micro-climatic challenges of heat in summer and cold in winter. To test the flexibility of our approach, we considered two different proposals for SCM that have been under consideration by the local government—*Flood scenario 01: ‘Land subject to inundation’,* and *Flood scenario 02: ‘Road-based integrated water management’.*

The overall test case application process involved 11 steps:

1. Site model construction (ensemble simulation) (see Section 6.1);
2. Application of the solar preservation model (see Section 6.2);
3. Extension of the solar preservation model to include floods (see Section 6.3);
4. Procedural presence–absence multi-conditions maps (floods, solar preservation and shade) (see Section 6.4):
  - *Flood scenario 01: ‘Land subject to inundation’*;
  - *Flood scenario 02: ‘Road-based integrated water management’*;
5. Develop tree selection criteria based on the four co-conditions (see Section 6.5);
6. Create sets of proxy-object scatters over the street network categorised by width and orientation (see Section 6.6);
7. Adjustment to achieve summer shading criteria (see Section 6.7);
8. Visualisation of streetscape designs—multi scale visual impact renderings (see Section 6.8);
9. Fit indicative tree species suitable for the conditions criteria (see Section 6.9);
10. Visualising tree selections for iterative storm-water management scenarios (see Section 6.10);
11. Light level analysis for expressing summer shading outcomes (see Section 6.11);

## 6. Results: Application to a Complex Urban Renewal Test Case Site

### 6.1. Site Model Construction (Ensemble Simulation)

The test case site, Arden-Macaulay, a major urban renewal project with distinct flooding as well as micro-climatic challenges of heat in summer and cold in winter, is located in the inner north of Melbourne, Australia. In this development, the proposed ‘street wall’, (or zero lot development), will have a substantial impact on both summer shade and winter overshadowing of streets. Our first requirement was to quantify to where, and at what time of day, the proposed built form would overshadow the streets in winter, so as to ascertain when solar preservation would be possible.

**Figure 4:**
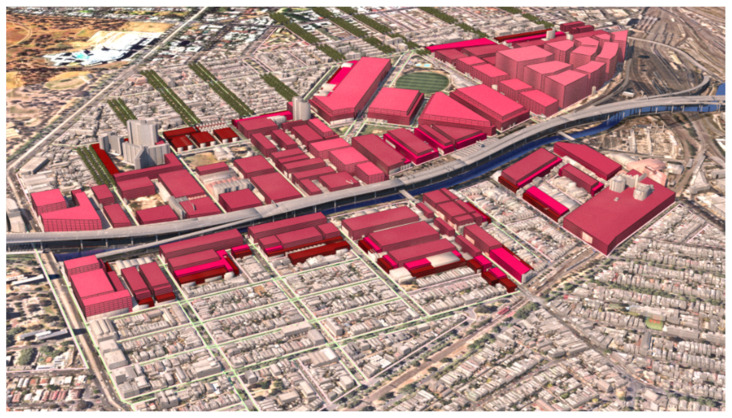
Massing model of maximum building envelopes regulated in the C190 amendment: dark red, 10.5 m interfacing residential areas; pale pink, height restrictions up to 60 m. Image by authors using multi-criteria street design decision support modelling approach in Autodesk™ 3ds Max™.

We constructed a 3D digital model of the site, spatialising the written proposed building envelopes (built-form) outlined in the Arden-Macaulay Structure plan and the C190 planning scheme [94,95]. The dominant proposed condition was twenty-metre-wide streets with simple (vertical façade), six storey buildings (20 m height) (canyon ratio of 1 H:1 W), streets oriented (bearing N 7° E) [94]. The proposed maximum built form model was procedurally generated and therefore extremely adjustable, allowing for changes or alternative scenario building regulations (Figure 4).

We integrated high-quality existing 3D building models of the LGA of City of Melbourne combined with land parcel maps and street centre lines from PSMA Australia Limited 2016, street casements from Vicmap Property 2017, and water bodies and flood extent from Melbourne Water corporation and City of Melbourne Urban Forest data, which are available to researchers through the Australian Urban Research Infrastructure Network (AURIN). The models of the currently existing site buildings adjacent to the renewal precinct were supplied in ‘geo chunks’ (portions of the municipality that do not necessarily align with traditional suburb boundaries or statistical area divisions. This is the term used by the municipality) for each suburb within the Local Government Area (LGA). These were amalgamated into a single model from which the focus area could be selected. As the site has several heritage-registered buildings, we began by classifying the building models into three groups (retain/demolish/partially demolish) (Figure 4). Heritage registered buildings remain as single or double storey and will not, therefore, have the same overshadowing impact on the street as the adjacent new built form.

### 6.2. Application of the Solar Preservation Model

Using a solar metric sun system allows accurate building shadow renderings to be produced for any time of the day and day of the year. We rendered summer and winter shadow maps for the three primary pedestrian peak times associated with mixed used development: morning 8:00–9:00, mid-day 12:00–13:00, and afternoon 15:30–17:30 [96]. From the rendered shadow maps we ascertained that in winter, the morning and afternoon peaks would be entirely overshadowed, and therefore selected the mid-day peak as the focus time for solar preservation. Conversely, in summer, this mid-day peak coincides with the highest daily UV levels (In Victoria in summer the UVI is regularly above 12–14 at midday [97]) and little building shade on footpaths and cycle paths. Figure 1 shows a detailed view of an intersection at 12:30 (adjusted for daylight savings) in summer (RIGHT) and winter (LEFT). We then used the model to render out an accurate precinct-wide, head height, mid-day mid-winter ‘solar preservation (overshadowing) map (shadows only without geometry) using the same process as described in [57]. This map was simplified to a black and white binary image and re-projected onto the ground plane of the model.

### 6.3. Extension of the Solar Preservation Model to Include Flood

**Figure 5:**
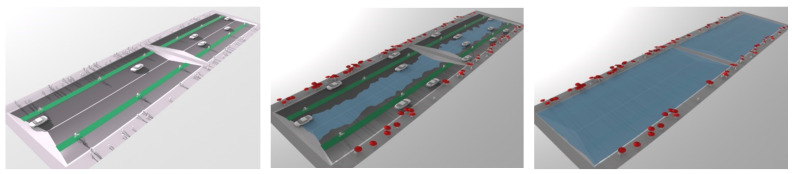
Street casement graded to hold storm water in the road casement in non-flood to major flood events. Image by authors using 3ds Max.

The next step was to bring the storm water management plans and the building shadow plans into a single comparable georeferenced raster map format. This required 2D renderings of storm water management simulations output from external flood modelling programs as described by Tomkins and Lange [98]. This is an extremely efficient method of integrating storm water management scenarios into urban design decision-making, as it allows for extensive iteration and avoids errors associated with re-simulation of water management scenarios over imported triangulated irregular networks (TIN) by ‘non-water’ professionals [98]. One of the primary proposals for stormwater management on this site is the use of street casements for storm water detention (shown in Figure 5, without the associated tree planting). Trees in these systems may require a number of highly specific tolerances, which would need to be considered at the implementation level. For this pilot demonstration study the focus was on tree function in two aspects of storm water management: The slowing of initial rates of overland flow through canopy rainfall interception, and clearing water holding capacity in bioretention systems through seasonally continuous, high rates of evapotranspiration [50].

### 6.4. Procedural Presence–Absence Multi-Conditions Maps (Flood, Solar Preservation and Shade)

**Figure 6:**
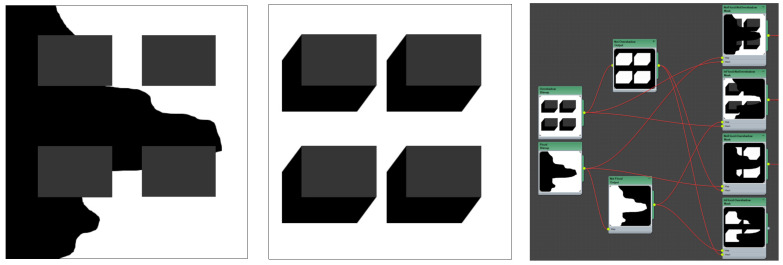
Screengrab showing an indicative intersection detail with four buildings (in grey) and flood map (black); MIDDLE shows the winter building overshadowing map at 12:00 (shade in black); RIGHT shows how these maps are brought together to generate four ‘presence–absence’ conditions maps. Image by authors using Autodesk™ Slate Editor.

By using this mapping technique, both built form and storm water management proposals can be brought together and considered simultaneously in an integrated and spatially explicit way. As a demonstration, Figure 6 shows an indicative stormwater management plan (flood map) overlayed with an indicative solar preservation map and how they are brought together to create four distinct *procedural presence*–*absence* conditions maps. The intersections of different co-conditions result in specific criteria for tree planting (see Table 1): in a flood area/not in flood area and winter shadow/winter sun. The initial flood and overshadowing maps are simple to replace, as ‘inverse copies’ of each map directly connect into an invert operation, followed by a ‘masking operation’, procedurally updating the areas of each co-condition (see RIGHT, Figure 6).

In the site model, each conditions map is accurately projected onto a ground surface plane (using a process known as ‘UVW mapping’) (three-dimensional material texture mapping; the third dimension (W) allows textures to wrap onto complex geometry with three-dimensional surfaces). The four conditions can be combined into a single material by assigning them different ‘material ID’s. This procedural process allows any changes to the flood map (such as extent) or the overshadowing map (such as height of the street wall, time of day/year) to be updated by simply replacing or plugging a different storm-water management scenario (flood map) or shade map into the initial map slots.

**Figure 7:**
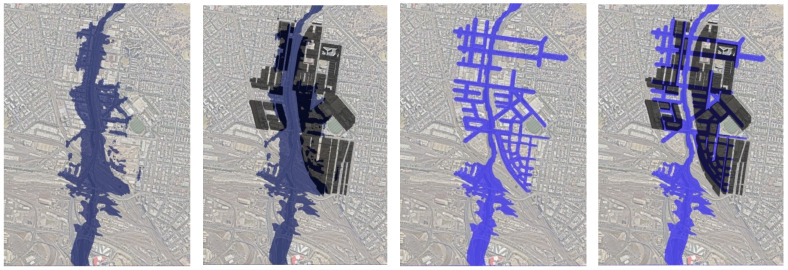
LEFT Map of Flood scenario 01: ‘Land subject to inundation’ flood zone as modelled by Melbourne Water 2016 with no infrastructure changes and overlayed on the 12:30 mid-winter building overshadowing map RIGHT map showing Flood scenario 02: ‘Road-based integrated water management’ Surface storm water infrastructure within the street casement overlayed with the 12:30 mid-winter building overshadowing map.

In the site model we tested two flood scenarios against the 12:30 mid-winter building overshadowing condition, demonstrating how the model allows iteration of intensively different proposals. We created two black and white images, one for two potential storm water management strategies (referred to as flood scenarios) described below and shown in Figure 7.

***Flood scenario 01: ‘Land subject to inundation’***: No new control measures are implemented for the current extent of 1 in 100 year flooding of the site (also known as the planning overlay for ‘Land Subject to Inundation (LSIO) in the Melbourne Planning Scheme) [99] (LEFT Figure 7 with and without the solar preservation map overlay).

***Flood scenario 02: ‘Road-based integrated water management’***: Flooding is controlled using road-based integrated water management, which utilises the street casement as detention ponds (RIGHT Figure 7 with and without the 12:30 p.m. winter building solar preservation map overlay), derived from a three-dimensional TIN mesh of the road-based storm water management proposal.

**Figure 8:**
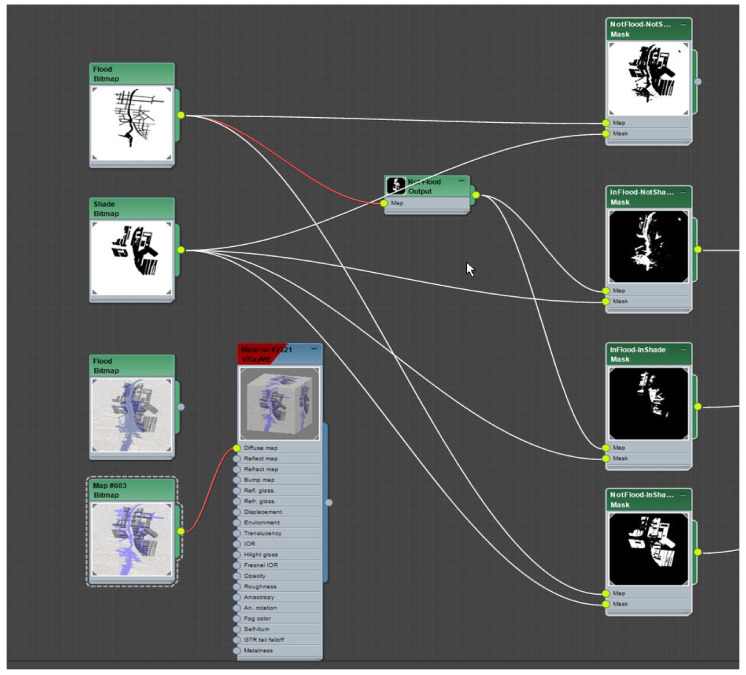
Conditions masking maps used to drive the suppression and occurrence of tree models within the proxy-object scattered along streets across the entire precinct. Image by authors using Autodesk™ Slate Editor.

To create binary co-condition presence–absence maps (Figure 8), we brought these flood condition maps together with the building solar preservation map for 12:30 p.m. in winter using the material mapping technique described above.

### 6.5. Develop a Tree Selection-Criteria Based on the Four Co-Conditions

We then used the four co-conditions to establish a tree selection criteria matrix. In the co-condition of ‘winter sun preservation’ and ‘in flood’, species need to be deciduous to allow maximum light penetration in winter and have qualities related to storm water management such as a high transpiration rate or rainfall canopy storage capacity [50,100]. In the co-condition of ‘overshadowed at midday in winter’ and ‘not in flood’, species can be evergreen to maximise interception of rainfall, but may also need to be tolerant of dry conditions if additional water resources are not available in dry weather periods (see Table 2). These co-conditions and their complexities are discussed later. Using this spatial method to identify exact locations where specific criteria need to be met ensures maximal use of evergreen trees to meet functional criteria for storm-water management whilst simultaneously maximising preservation of winter solar access on streets.

### 6.6. Create Sets of Proxy-Object Scatters over the Street Network Categorised by Width and Orientation

As described in detail in Langenheim et al. [57], streets can accommodate trees in a very limited number of positions, the most common of which are: At the property boundary, at the curb, between carparking spaces, and within a central median. The potential positions for trees on a given street is predicated on street width and property boundary condition (setbacks or no setbacks). Over offsets of vectors representing street centrelines, categorised into different widths and orientations, we placed proxy-object tree model scatters (lines of high polygon modelled tree objects) representing species that meet the criteria for each co-condition. This process resulted in four trees placed into each possible tree location on every street. A corresponding *conditions presence*–*absence map* was then connected to each line of tree models.

**Figure 9:**
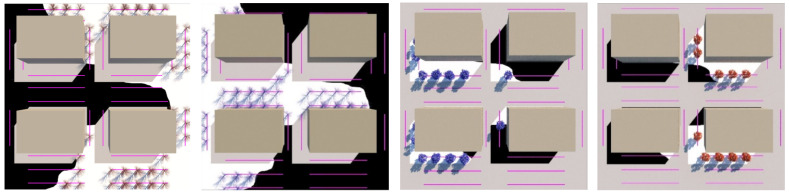
Shows tree models suppressed in the black sections of the map. In this map, black represents areas that are both overshadowed by buildings and affected by floods. The tree models are then only allowed to occur in the white areas representing conditions where winter solar access is possible, and flooding does not occur. The three other condition maps control the suppression or occurrence of trees in each proxy-object tree scatter. NOTE: due to the use of the ‘masking operation’, beige also works as a black suppression area. Image by authors using multi-criteria street design decision support modelling approach in Autodesk™ 3ds Max™.

**Figure 10:**
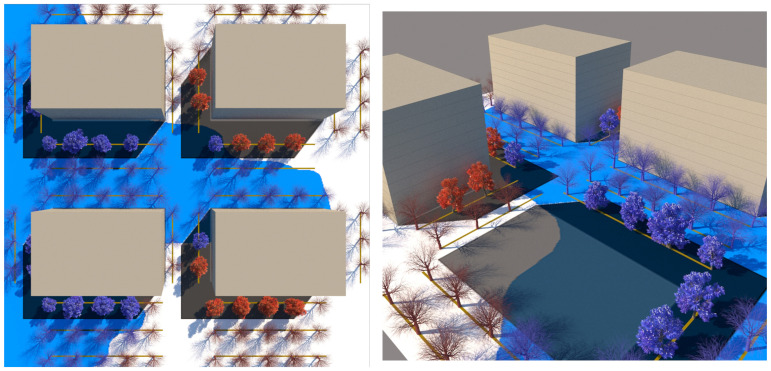
LEFT shows the four map-controlled proxy-object tree scatters with blue deciduous models occurring in flood + solar preservation, red deciduous models occurring in dry, solar preservation, blue evergreen models in flood overshadowing, and red evergreen models in dry overshadowing. Note: flood is depicted in blue for clarity. RIGHT shows a perspective view of the intersection model (with a foreground building mass removed). The models are colour- and form-coded to their respective condition. These models are now ready to be adjusted to the requirements of solar exposure protection (crown-form, canopy dimensions, position and spacing). Image by authors using multi-criteria street design decision support modelling approach in Autodesk™ 3ds Max™.

**Figure 11:**
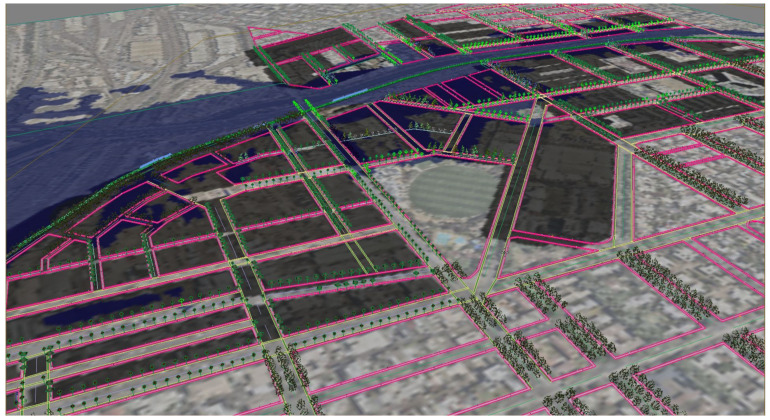
Aerial view screen grab the presence–absence mapping tree choice approach applied to the whole of the Arden-Macaulay site study scenario 1 ‘Land subject to inundation’ showing tree geometry model species placement responding to flooding conditions (in flood or not in flood) and shade conditions (in shade or not in shade). Tree models are displayed as simplified to point-clouds and simple shapes for computational efficiency. Image by authors using multi-criteria street design decision support modelling approach in Autodesk™ 3ds Max™.

The lines of proxy-object tree model scatters ‘sample’ the pixel colour of their underlying conditions *presence*–*absence map* to govern if they will be suppressed (over black) or allowed to occur (over white). Tree models on three of the four concurrently occurring tree models will be suppressed, leaving only a single tree model visible per tree position available. Figure 9 shows the model in plan and Figure 10 shows perspective views, with lines of trees along the streets, suppressed over black areas (where their co-condition does not occur), and allowed to occur over white (where their co-condition is present). Figure 11 shows the model working at the whole site scale, with tree model suppression/occurrence controlled by their whole-site corresponding *presence–absence map*.

### 6.7. Adjustment to Achieve Summer Shading Criteria

The next part of the process allowed for adjustment of the tree locations to meet positioning requirements for optimal summer shade provision to the streetscapes. This process required the fitting of specific tree species to each of the co-conditions to inform geometric proportions and growth habit requirements of trees to be planted in the precinct. For instance, the co-condition of ‘allow winter sun + does not occur in a place that requires high rates of transpiration’ would best suit a species that is deciduous, and given Australia’s climatic conditions, is tolerant to extended periods of drought. The process of fitting species to the resulting conditions brought into focus how restrictive even this limited number of performance criteria is for tree selection. For instance, the condition of ‘in flood, not shade’, would best be fitted to a species that constantly transpires to assist in clearing retention systems and that is also deciduous to preserve available winter sun to the street. This combination of characteristics is likely a biological anomaly as deciduous trees do not transpire while dormant. For this condition we selected the indicative *Nyssa sylvatica* (Black Tuplo tree) as it is both deciduous and has high transpiration rates. This condition is also uncommon in Melbourne with its weather patterns of long dry periods, and thus species expected to survive and perform as needed in these locations would require access to additional water resources. In a real-world application, species ‘fitting’ would require coordination with tree managers or municipal arborists and would include planning for irrigation infrastructure.

Once indicative tree species were fitted to the tree selection criteria, their geometric proportions were used to inform the position of the property boundary offset lines, into the best possible tree location available in each category of street (width and orientation) for producing summer shade at mid-day over the cycle path and or footpath. Due to the building development proposal (street wall), options for the position of the trees were limited to two, either in the footpath or in the road reserve.

### 6.8. Visualisation of Streetscape Designs-Multi Scale Visual Impact Renderings

The next part of the process was the output of visual material for decision-support and community consultation forums. Full visual impact renders (also known as ‘beauty rendering’ in the CGI industry) can be output at multiple scales from the model.

To allow the building models generated from the planning guidelines to appear less abstract (realistic enough for understanding the scale of development with apparent floor levels and windows) we used two scripts: ’Vu-normalise spline’ to divide the spline geometry evenly to give the appearance of façade window and door articulation, and Tom Hudson’s ‘Greeble’ (Tom Hudson’s Greeble plugin to 3ds Max http://max.klanky.com/plugins.htm, accessed on 1 February 2018) to simulate indicative mixed-use building forms.

### 6.9. Fit Indicative Tree Species, Suitable for the Conditions Criteria

In the previous iteration of this approach described in Langenheim et al. [57], we first used simplified ‘tree geometry models’ (simple 3D lollipop models representing growth dimensions of selected species) to test for the most suitable location to achieve shading criteria; however, in this case we were able to skip this step and fit directly with the indicative tree types in Table 2, with visually realistic models of those species selections.

In this step we fitted either a procedurally grown or a library asset ‘indicative tree species’ suitable for the four conditions, supplanting the initial ‘tree type models’ as shown in red and blue in Table 3. In this instance, we used three commercial 3D tree models available from the Xfrog™ library, and one we built ourselves procedurally (using ExLevel GrowFX™), as this species was not available from any commercial tree libraries.

### 6.10. Visualising Tree Selections for Iterative Storm-Water Management Scenarios

**Figure 12:**
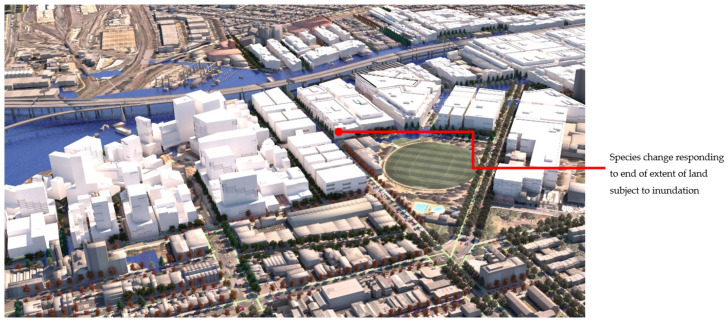
Rendering, aerial view under flood condition for scenario (1), ‘Land subject to inundation’. This results in changes of species that may cross roads or alter with no physically apparent infrastructure. Image by authors using multi-criteria street tree design decision support modelling approach in Autodesk™ 3ds Max™.

**Figure 13:**
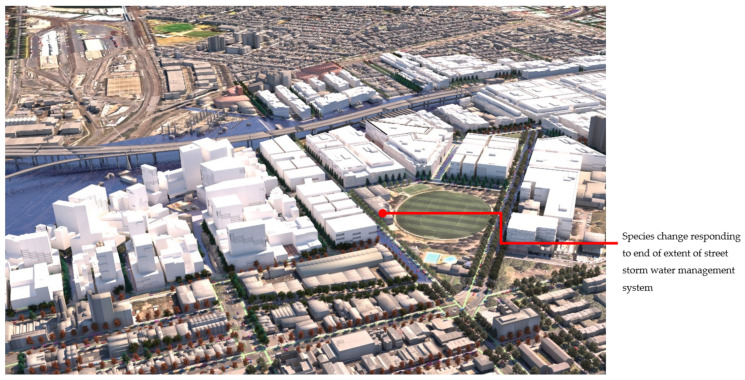
Rendering, aerial view north-west under flood condition for scenario (2), road-based integrated water management. Image by authors using multi-criteria street tree design decision support modelling approach in Autodesk™ 3ds Max™.

**Figure 14:**
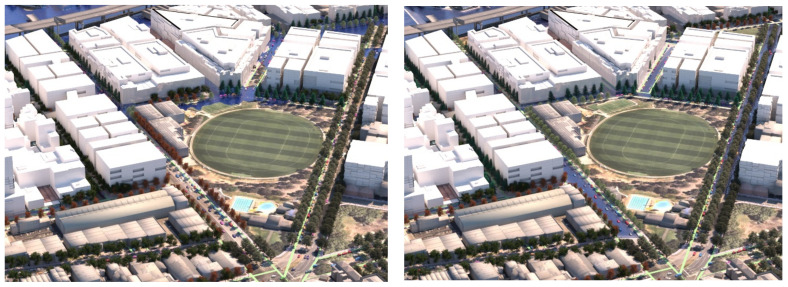
Detail: LEFT Flood scenario (1) and RIGHT Flood scenario (2) showing species change responding to flood extent. Image by authors using multi-criteria street tree design decision support modelling approach in Autodesk™ 3ds Max™.

**Figure 15:**
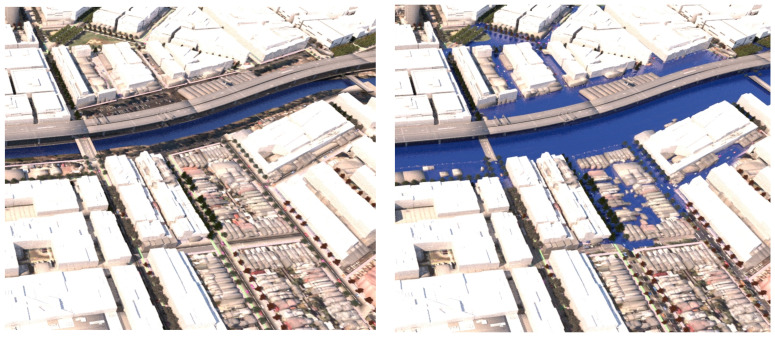
Detailed rendering, aerial view looking east for scenario (1) ‘Land subject to inundation’ LEFT under dry conditions and RIGHT under flood conditions. Image by authors using multi-criteria street tree design decision support modelling approach in Autodesk™ 3ds Max™.

**Figure 16:**
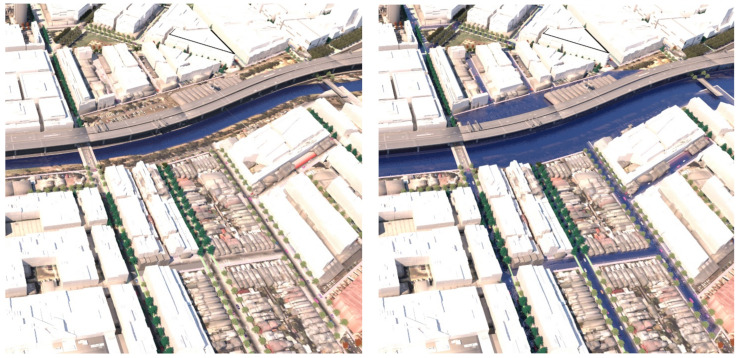
Detail rendering, aerial view looking east for scenario (2) road-based integrated water management LEFT under dry conditions and RIGHT under flood conditions. Image by authors using multi-criteria street tree design decision support modelling approach in Autodesk™ 3ds Max™.

Figure 12 shows **flood scenario 1**: ‘*Land subject to inundation’* in flooded condition, at precinct scale looking north-west. The lines of *Pinus strobus* (in flood/overshadowed condition) can be seen switching over to *Fraxinus pennsylvanica,* (red over-toned tree model) as the street runs past the oval and comes into the condition of dry + not overshadowed by buildings. Figure 13 shows how in **scenario 2:** *road-based storm water management* at this same point, the tree species switches instead from *Pinus strobus* to *Nyssa sylvatica*, responding to the underlying co-conditions map of the road-based detention basins, that continue through the length of the street. Figure 14 shows a comparison of the detailed area discussed on the left (**scenario 1**) and on the right (**scenario 2**). Figure 15 and Figure 16, show views of the model looking east, comparing the change in tree species occurrence and suppression responding to the two different flood scenario driver maps both in flood and in dry conditions. These images demonstrate the more regular changes in species in **scenario 2**, road-based storm water management, as flood conditions are more controlled than in **scenario 1**.

**Figure 17:**
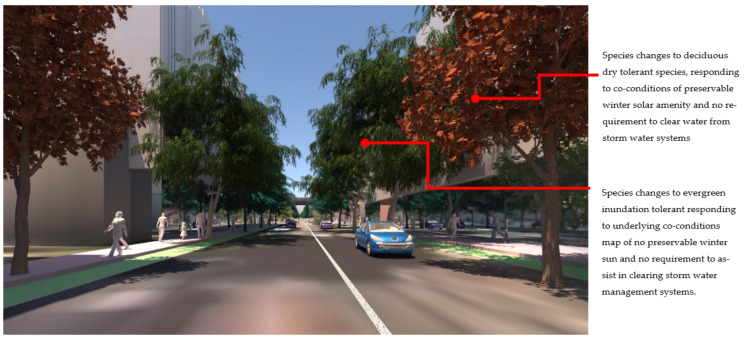
Macaulay Road view of Flood scenario (1) ‘land subject to inundation’ during dry weather. Several changes in tree species can be seen in the background responding to the underlying flood condition map, but with cross street symmetry. This image also shows the asymmetric tree positioning. Image by authors using multi-criteria street tree design decision support modelling approach in Autodesk™ 3ds Max™.

**Figure 18:**
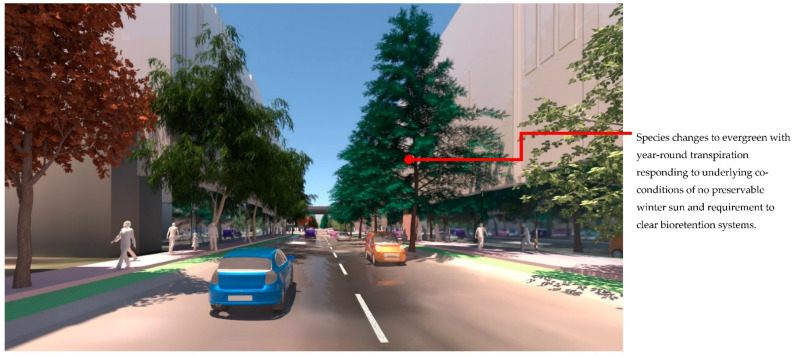
Macaulay Road view of Flood scenario (2) ‘road-based integrated water management’ during dry weather. In both schemes trees on the right-hand side are positioned in the road just beyond the bike path to achieve the summer active transport objective. Image by authors using multi-criteria street tree design decision support modelling approach in Autodesk™ 3ds Max™.

**Figure 19:**
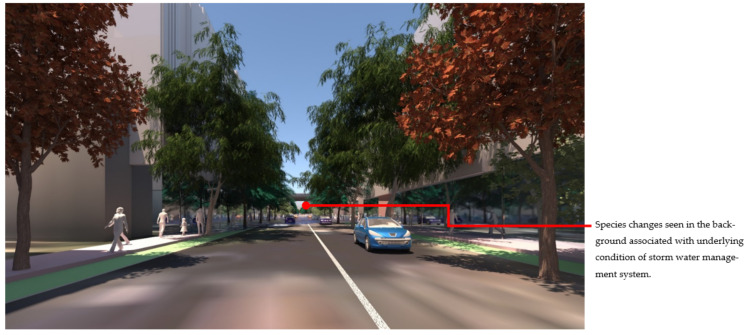
Macaulay Road view of Flood scenario (1) ‘land subject to inundation’ during flood (which can be seen in the background). The *Angophora costata* in the mid-ground occur over a symmetrical condition (dry and overshadowed in winter). Image by authors using multi-criteria street tree design decision support modelling approach in Autodesk™ 3ds Max™.

**Figure 20:**
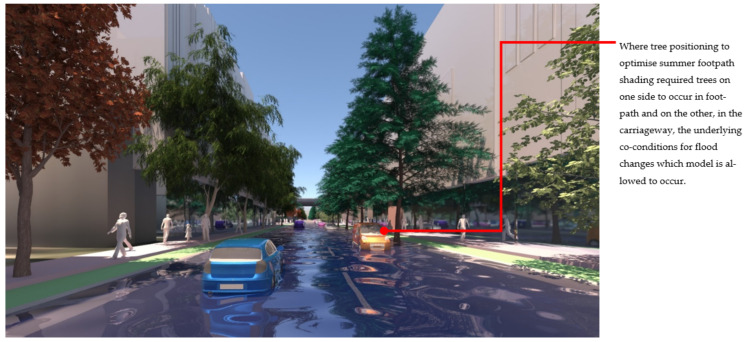
Macaulay Road view of Flood scenario (2) ‘road-based integrated water management’ during flood. Trees in this scene do not occur over symmetrical conditions. Trees planted in the road (right hand side) experience wet and overshadowed conditions and thus the species switches to *Pinus strobus*, while trees in the footpath still experience dry overshadowed conditions and thus remain as *Angophora costata*. Image by authors using multi-criteria street tree design decision support modelling approach in Autodesk™ 3ds Max™.

**Figure 21:**
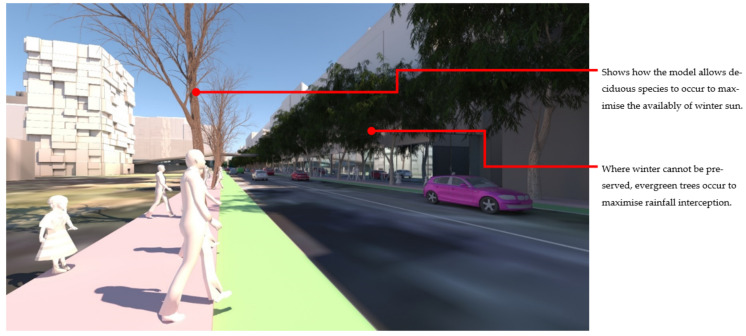
Image shows a winter view of Macaulay Road. On the righthand side the evergreen trees (*Angophora costata*) occur in the (dry/overshadowed condition), and on the left-hand side, where winter solar access to the footpath can be preserved, deciduous species occur (wet or dry tolerant depending on the flood scenario). Image by authors using multi-criteria street tree design decision support modelling approach in Autodesk™ 3ds Max™.

At the streetscape level, the visual impact of these different storm-water management scenarios can be assessed in detail. Figure 17 shows a view down Macaulay Road of the streetscape outcome during flood conditions for scenario (1) and Figure 18 shows the same for scenario (2). In flood scenario 1, patches of symmetrical planting occur such as can be seen in Figure 17 where the *Angophora costata* model occurs in response to the map of a dry and overshadowed condition. However, in flood scenario (2), where flood conditions continue through the length of the street, trees planted in the road are different from those in the footpath as they must tolerate inundation. In scenario (2) there is more longitudinal symmetry, with species changes most affected by their position in the road to achieve the summer shading objective. Figure 19 and Figure 20 show the same views of Macaulay road but in flooded condition. These images show how the different flood scenarios affect the tree-scape outcomes. Figure 21 shows Macaulay Road during winter, demonstrating the system allowing evergreen trees to occur in dry winter overshadowed conditions but deciduous species occurring where winter sun was able to be preserved.

### 6.11. Light Level Analysis for Expressing Summer Shading Outcomes

**Figure 22:**
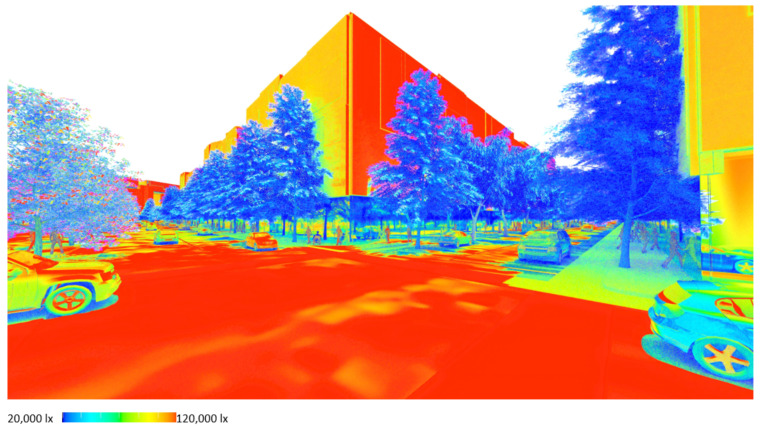
Rapid lux level lighting calculation for quick visual shading analysis at the streetscape scale. Image by authors using multi-criteria street tree design decision support modelling approach in Autodesk™ 3ds Max™.

**Figure 23:**
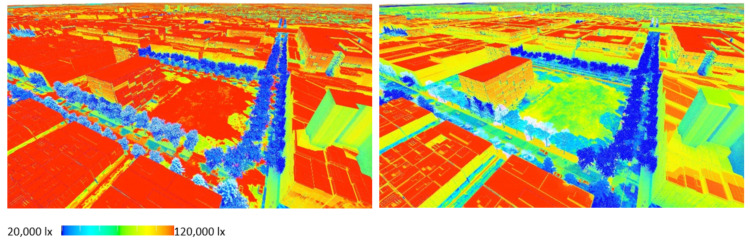
Rapid lux level lighting calculation for winter (left)/summer (right) seasonal comparison. Image by authors using multi-criteria street tree design decision support modelling approach in Autodesk™ 3ds Max™.

Finally, Figure 22 and Figure 23 show the possibilities of using a rapid lux level lighting function available through the rendering system for visualisation and analysis of the depth of shade provided on bike- and footpaths in summer. The lighting analysis can be done at both the precinct and streetscape scales, giving a quick indication of amounts of shade that can be numerically analysed where required. This form of visualisation could be instrumental in demonstrating the shade impact of tree selections in community consultation environments.

## 7. Discussion

In this paper we presented a visual-functional street treescape design decision approach for moderating between conflicting tree criteria for stormwater control, sunlight and visual impact, using ensemble simulation, co-conditions presence/absence mapping and 3D high polygon tree modelling. This approach is particularly useful in the initial stages of design of urban renewal sites where limitations of existing street networks and flood conditions must be addressed through integrated design of green infrastructure.

The approach outlined in this paper treats trees, which have previously been considered an expensive ornamental element in streets, as critical urban infrastructure. By integrating our non-siloed approach to early stages of a treescape design process, it is possible to make early decisions about their placement, species and form, all crucial traits for meeting sustainability and environmental goals in cities [27,101].

Our non-siloed approach departs from traditional street tree decision making, where trees are often the last consideration, fitted into spaces left between driveways, above and below ground utilities and traffic visibility requirements, and where species are selected on the basis of tradition and other visual criteria such as neighbourhood character or resident preference by putting them at the forefront of street design.

The aspects of street tree selection the approach can work with are the preservation of streetscape solar access in winter, ‘optimisation’ of shade in summer for cyclists and pedestrians, and maximisation of evergreen trees for flood adaptation. The environmental performances required of trees in urban development for these criteria are often conflicting, meaning that conscious and deliberate choices and trade-offs will need to be made that are informed by spatial constraints, flood modelling outputs, built form regulations and accurate solar conditions. In addition, the approach allows for the instantaneous update of tree quantities required for each co-condition type.

The visualisation output from this decision model demonstrates how tree decisions based on these functional criteria may lead to quite different aesthetic experiences on streetscapes from the traditional symmetrical plantings of the 19th century, though some symmetries still occur. In scenario 1: Land subject to inundation, several changes occur along the length of the street but are often symmetrical across the street. In **scenario 2**: Road-based storm water management, the opposite occurs. Street tree species, responding to the controlled flood map for the road surface, remain relatively constant along the length of the street (changing only in response to light conditions) while across the street, due to the different tree positioning for achieving the summer shading objective, species change. This visual departure is particularly apparent in **flood scenario 01**, where the underlying co-conditions cause trees to change type, mid street section (see Figure 20). If tree selection for flood moderation and minimising winter overshadowing of streetscapes were implemented, the visual quality of the resulting streets will differ from traditional streetscapes, where placement and species are typically arranged symmetrically, and may be reminiscent of the designs of naturalist Frederick Law Olmsted [102].

In this study we constructed 3D building models from written guidelines, which raised questions about the environmental performance of these future buildings, their impact on the quality of the streetscape and how they interact with provision of space for trees and flood infrastructure. By spatialising these written building form regulations, we were able to bring these three aspects of urban renewal into simultaneous, equitable and iterative consideration, potentially allowing the design of green infrastructure to inform building proposals in future cities, rather than post-operatively applied as a last moment consideration.

Once the model is constructed it is capable of rapid and flexible iteration, resulting in significant time savings when responding to building proposals or flood projection changes. The input conditions maps make it simple to change and immediately update the streetscape design, including total number of each species, thus informing tree growers of the quantity of future trees required to survive and thrive in conditions that deliver green infrastructure. The selection of tree species for their specific flood moderating functions is complex and enabling preliminary criteria through this modelling approach should have a profound impact on grower’s species selections.

### Limitations and Future Research Directions

While the modelling presented in this study expands on prior work and includes mutliple factors not previously considered together simulatinaiously, there are many other factors that could be considered and potentially integrated into the system in the future. This model was set up with static trees as objects rather than living, growing subjects. In future research, other aspects that impact tree growth could be added such as soil conditions and light level growth impact, as well as age of trees and expected growth rates to show change over time as sapplings grow to full-sized trees.

There may be potential for the model to also add the calculation of carbon capture, as well as the potential for improving air quality. Lower vegetation might also be considered in the model, such as low-level shrubs and grasses used in rain gardens, which can be used to clean storm water coming from roads, flitering out heavy metals before entering waterways [103].

Another aspect that is worthy of consideration is the compatibility of urban street trees with the promotion of active travel modes including walking and cycling [104]. While the provision of street trees can enhance the active travel experience [22,105], the placement of trees must be considered carefully so as to not obstruct the movement of cyclists (when planted in road reserve areas) and pedestrians when in or close to footpaths if trees become to large and block or disrupt the footpath surface.

## 8. Conclusions

This iterative data feedback process could be adapted to different environmental conditions and criteria in different locations. Adoption of the approach has the potential for improving design processes and integrating the science of nature-based solutions into governance and land use planning for urban renewal. Further to this, the model can be used for testing the integrated performance of other systems such as bike path locations and safety measures in flood conditions and has the potential to fulfil the role that urban design frameworks currently occupy.

Our multi-criteria modelling approach makes a significant contribution to the arsenal of urban designers, planners and landscape architects in their pursuit of smarter streetscape design decisions that respond to complex spatial, cultural and climatic urban challenges, and maximises the benefits of trees in green infrastructure.

## Figures and Tables

**Table 1:** The four co-conditions informing tree selection criteria when overlaying the flood and overshadow maps.

	IN FLOOD	NOT FLOOD
WINTER SUN	Solar access preservation + water regulation	Solar access preservation + dry
WINTER SHADOW	No solar access preservation + water regulation	No solar access preservation + water regulation

**Table 2:** Indicative tree types needed for each co-condition. Images by authors using Autodesk™ 3ds Max™ with Xfrog™ ExLevel GrowFX™ trees.

		IN FLOOD		NOT FLOOD
WINTER SUN	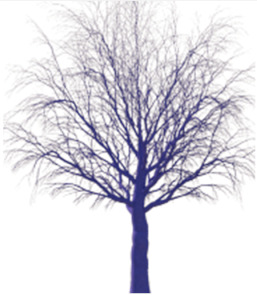	Deciduous flood tolerant	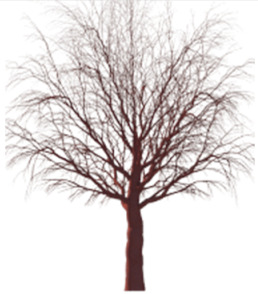	Deciduous dry tolerant
WINTER SHADOW	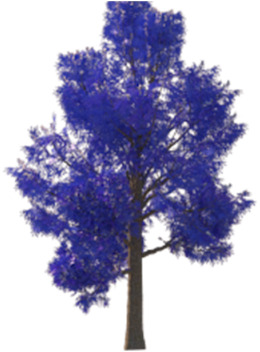	Evergreen flood tolerant	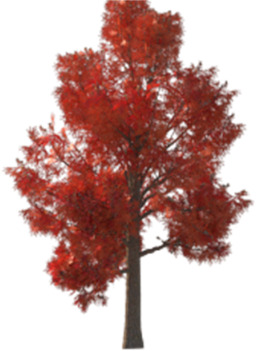	Evergreen dry tolerant

**Table 3:** Indicative species selection to respond to flooding conditions (in flood or not in flood) and shade conditions (in shade or not in shade). Images by authors using Autodesk™ 3ds Max™ with Xfrog™ ExLevel GrowFX™ trees.

Condition	Not Flood/ Not Shade	In Flood/ In Shade	In Flood/ Not Shade	Not Flood/ In Shade
Requirements	Dry/ Deciduous	Wet/ Evergreen	Wet/ Deciduous	Dry/ Evergreen
Indicative species	*Fraxinus pennsylvanica* *(Ash)*	*Pinus strobus* *(White Pine)*	*Nyssa sylvatica* *(Tulepo)*	*Angophora costata* *(Smooth-barked apple)*
Notes	Many trees are suitable for this condition *Red over-toned for clarity in renders*	Would require additional water resources in dry conditions	Would require additional water resources in dry conditions	A species that has better canopy interception qualities might be better here [100]
	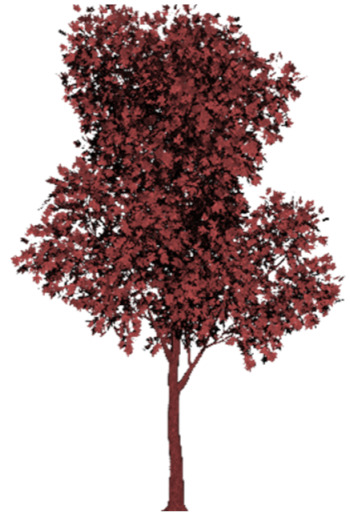	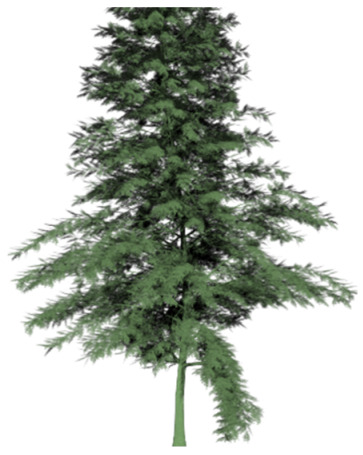	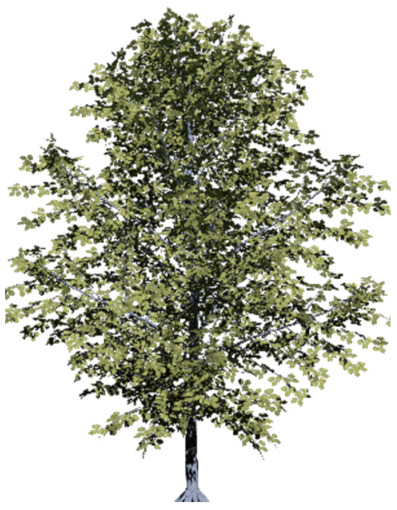	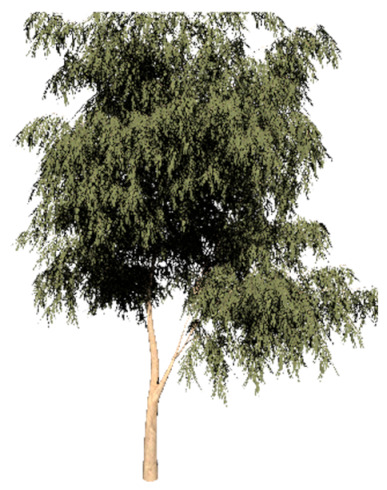

## Data Availability

Not applicable.
